# Perceived Access to HIV Prevention Services Amidst the COVID-19 Pandemic Among Men Who Have Sex with Men (MSM) and MSM Sex Workers in France, Russia, and Türkiye

**DOI:** 10.1007/s10508-024-03027-9

**Published:** 2024-11-05

**Authors:** Kristopher J. Jackson, Tadhg Sullivan, Sean Howell, Alex Garner, Glenn-Milo Santos

**Affiliations:** 1 UCSF Center for AIDS Prevention Studies, University of California, 550 16th Street, 3rd Floor, San Francisco, CA 94158 USA; 2 Division of Infection, University College London Hospitals NHS Foundation Trust, London, UK; 3 LGBT Foundation, San Francisco, CA USA; 4 MPact Global Action, Oakland, CA USA

**Keywords:** Men who have sex with men, COVID-19, HIV prevention, Sex work, PrEP, Sexual orientation

## Abstract

This study examined the association between self-identification as a sex worker (SW) and perceived access to pharmacologic and non-pharmacologic HIV prevention methods among MSM in France, Russia, and Türkiye amidst the COVID-19 pandemic. Globally, 17,250 MSM recruited through a geosocial networking smartphone application completed the COVID-19 disparities survey, which was administered between October and November 2020. Approximately 38% of survey respondents were identified as living in France (n = 1269), Russia (n = 3882), and Türkiye (n = 3141) at the time of survey completion. Given the diverse sociodemographic factors and attitudes toward both MSM behavior and commercial sex work in these countries, we conducted a secondary analysis of survey data exploring the relationship between SW status and perceived access to pharmacologic and non-pharmacologic HIV prevention methods during the COVID-19 pandemic. Among respondents in Russia and France, MSM SW status was associated with a reduction in perceived access to condoms/lubricants (*p* = .001 in Russia, *p* < .001 in France). MSM SW in France were less likely to report never using PrEP as compared to non-SW peers (RR = 0.40, *p* = .005). Our findings highlight the disparities in access to HIV prevention for MSM SW living in these three countries during the COVID-19 pandemic. Based on our findings, COVID-19 may have exacerbated pre-existing inequities in HIV prevention among populations experiencing intersecting stigmas.

## Introduction

Among the many interruptions to healthcare services during the COVID-19 pandemic, routine primary care and preventative medicine services were disproportionately impacted (Lim et al., 2021). In many jurisdictions, these disruptions resulted in diminished access to sexual health services. To date, it is unclear whether the reduced incidence of sexually transmitted infections observed in multiple communities during the COVID-19 pandemic is a result of reduced transmission rates or decreased screening rates (Ivarsson et al., 2022; Sentis et al., 2021; Centers for Disease Control & Prevention, 2022).

COVID-19-associated interruptions to HIV/STI prevention and treatment may have had a disproportionate impact on members of the global MSM community. In a recent international survey of sexual and gender minority men (*n* = 12, 210) conducted by Rao et al. (2021), more than half of respondents reported diminished access to HIV testing and diminished access to HIV pre-exposure prophylaxis (PrEP) due to the COVID-19 pandemic. The authors also found that approximately 20% of survey respondents living with HIV reported interrupted access to HIV care. For MSM sex workers, the health risks associated with reduced access to sexual health services may have been exceptionally worrisome given the occupational hazard(s) associated with their work and their reliance on consistent access to healthcare services and support networks.

As members of an informal labor market, most sex workers were excluded from COVID-19-related governmental assistance programs and/or unemployment benefits. As a result, sex workers were forced to make difficult decisions about whether to continue seeing clients in person amidst government lockdowns and stay-at-home orders. According to UNAIDS (2014), sex workers are *twelve times* more likely to acquire HIV than the general population (UNAIDS, 2014). Further, MSM sex workers are six and a half times more likely to be diagnosed with a sexually transmitted infection (STI) than their female-identifying peers (Verhaegh-Haasnoot et al., 2015). While there is evidence to suggest that the COVID-19 pandemic caused service interruptions to sexual and reproductive health services globally (Mukherjee et al., 2021; Sanchez et al., 2020; Xiridou et al., 2021), less is known about the impact of these service interruptions on the lived experiences of MSM sex workers during the COVID-19 pandemic.

Given the illegal nature of sex work services in most jurisdictions, sex workers remain a non-traditional workforce underrepresented in most occupational health research. To describe the effects of COVID-19 on members of the global MSM sex work community, one must consider the context in which sex workers were living and working during COVID-19—a context that varied considerably between jurisdictions with respect to school and workplace closures, public gathering restrictions, stay-at-home orders, and travel bans (Hale et al., 2020).

Using data collected as part of a large-scale global COVID-19 disparities study (Santos et al., 2022), we describe and compare MSM sex workers and MSM community members’ perceived access to HIV prevention tools in three countries during the height of the COVID-19 pandemic. Our three study countries, France, Russia, and Türkiye, differ considerably in terms of COVID-19 response. However, these three countries also differ considerably in terms of LGBT rights, and the legal status of commercial sex work.

In the forthcoming sections, we present several of the most salient differences between the three study countries in each of these domains. This information is relevant because each nation’s policies and attitudes toward same-sex behavior and the legal status of sex work may affect a sex worker’s willingness to disclose high-risk behaviors to a healthcare provider. In jurisdictions where same-sex behavior is stigmatized or criminalized and sex work is illegal or marginalized, sex workers may routinely fear discrimination, harassment, or imprisonment. In these jurisdictions, sex workers may justifiably be reluctant to reveal these aspects of their identity to a healthcare provider. Conversely, in countries with legal protections for LGBTQ+ individuals and/or sex workers, some sex workers may be more willing to disclose sexual risk-taking behaviors to a healthcare provider. The social environment in which healthcare is rendered may have significant implications in terms of the ability to offer PrEP to individuals who might benefit from this HIV prevention method.

### France, Russia, and Türkiye: Variations in COVID-19 Response

As non-essential workers, sex workers were likely subject to stay-at-home orders and travel restrictions. Below, in Table 1, we summarize the work of Hale et al. (2021) describing the COVID-19 response in France, Türkiye, and the Russian Federation at the time of data collection.

**Table 1:** COVID-19 climate in France, Türkiye, and Russia at the time of survey administration (October-November, 2020)

COVID-19 Response	Country		
	France	Türkiye	Russia
School Closures	Required (at some levels)	Required (at some levels)	Required (at some levels)
Workplace Closures	Required for all but key workers	Required for some	Required for some
Cancellation of Public Events	Required cancellations	Required cancellations	Required cancellations
Restrictions on Public Gatherings	< 10 people	< 10 people	10–100 people
Stay-at-Home Restrictions	Required (except essentials)	Recommended	Recommended
Face Covering Mandates	Recommended	Required in some public spaces	No policy
Public Information Campaigns	Coordinated information campaigns	Coordinated information campaigns	Coordinated information campaigns
Public Transport Closures	None	Recommended closures or reduced volume	None
Restrictions on Internal Movement	Movement restricted	Movement restricted	No measures
International Travel Controls	Ban on travelers from high-risk regions	Screening required	Ban on travelers from high-risk regions
COVID-19 Testing Policy	Open public testing, including asymptomatic patients	Any individual with symptoms	Open public testing, including asymptomatic patients
Contact Tracing	Limiting tracing (only some cases)	Comprehensive tracing (all cases)	Comprehensive tracing (all cases)
Availability of COVID-19 Vaccine	None	None	None
Debt and Contract Relief	No relief	Broad relief	Narrow relief
Income Support	Covers > 50% of lost salary	Covers > 50% of lost salary	Covers < 50% of lost salary

Table contents adapted from Hale et al. (2021)

Notably, the COVID-19 responses adopted by all three countries collection are, in many ways, quite similar. Each mandated some form of school and workplace closure, initiated coordinated information campaigns, and maintained some form of international travel controls during the data collection period. With mandatory stay-at-home restrictions for non-essential workers, France appeared to maintain some of the strictest COVID-19 policies during the data collection period. Conversely, fewer restrictions were in place in the Russian Federation. For example, face coverings were not required by the Russian government when the COVID-19 disparities survey was conducted.

### France, Russia, and Türkiye: Differences in LGBT Rights

Because MSM sex workers engage in same-sex sexual behaviors, it is conceivable that local perceptions of homosexuality could influence MSM sex workers’ care-seeking behaviors. In Table 2, we describe the legal status of same-sex behavior in France, Russia, and Türkiye. This table is derived from data compiled by Equaldex, a collaborative knowledge base for the LGBT (lesbian, gay, bisexual, transgender) movement (Leveille, 2023). As shown in Table 2, the three study countries vary considerably with respect to LGBT rights. France appears to offer LGBT community members more rights and legal protections than Türkiye or Russia. Under Turkish law, homosexuality is not illegal, nor has it ever been outlawed or illegal. Despite this, the Turkish government maintains state-enforced censorship of LGBT “propaganda,” and same-sex marriage remains unrecognized. Most extreme, however, is the position on LGBT rights maintained by the Russian Federation. In Russia, LGBT community members are offered no governmental protections, and in some Russian provinces, homosexual behavior is punishable by death.

**Table 2:** LGBT rights in France, Türkiye, and Russia

	Country		
	France	Türkiye	Russia
Homosexuality	Legal	Legal	Varies by region^1^
Gay Marriage	Legal	Unrecognized	Not legal
Censorship	No censorship	State-enforced^2^	Fine as punishment
Discrimination	Illegal	Illegal	No protections
Employment Discrimination	Sexual orientation and gender identity	Sexual orientation only	No protections
Conversion Therapy	Banned	Not banned	Not banned
Military Service	Legal	Illegal	Don’t ask, don’t tell
1. Homosexuality is illegal in the province of Chechnya, punishable by caning on the first two offenses and execution after the third offense			
2. Pride parades and related public events are not allowed in Türkiye; Türkiye’s Ministry of Industry and Commerce requires online shops to mark LGBT-themed products with a minimum age of 18 +			

Table contents adapted from Leveille (2023)

### France, Russia, and Türkiye: Differences in the Legal Status of Sex Work

The legal status of commercial sexual activity varies slightly between the three study countries. Derived from data compiled by the Global Network of Sex Work Projects (), Table 3 highlights differences in the legal status of sex work in France, Russia, and Türkiye. Nearly all aspects of commercial sex work are illegal in all three nations except in Türkiye, where sex work is permitted in registered brothels. Turkish law maintains, however, that only unmarried women are allowed to work as sex workers in brothels. In France, buying sex is criminalized as opposed to selling sex. The opposite is true in Russia and Türkiye, where selling sex is considered illegal and buying sex is not a criminal act.

**Table 3:** Legal status of sex work in France, Türkiye, and Russia

	Country		
	France	Türkiye	Russia
Is selling sex criminalized?	No^1^	Yes^2^	Yes
Is buying sex criminalized?	Yes	No	No
Is organizing/managing sex work criminalized?	Yes	Yes, outside of brothels	Yes
Is sex work recognized as work?	No	No	No
1. Selling sex is legal; national-level soliciting laws were repealed when France criminalized clients. Municipal administrative laws remain in place criminalizing sex workers (e.g., prohibiting street sex work in certain jurisdictions)			
2. Sex work is legal only under certain restrictive conditions: sex workers can only work in a registered brothel and must be an unmarried woman who is also a Turkish citizen; working outside a registered brothel is illegal			

Table contents adapted from Global Network of Sex Work Projects (2021a, b, c)

### France, Russia, and Türkiye: Differences in PrEP Access

Globally, access to HIV PrEP across the World Health Organization European Region remains variable (ECDC, 2022). In 2016, France became the first European Union member state to make PrEP nationally available, covered by the French statutory health insurance system (Tassi et al., 2021). According to data obtained from the French National Health Data System, more than 42,000 individuals–more than 97% of whom were men/male-identifying–had initiated PrEP as of June 2021 (Billioti de Gage et al., 2022).

Access to PrEP is considerably different in Türkiye and Russia. In Türkiye, a generic formulation of PrEP has been commercially available since November 2021, but PrEP is not covered under the prescription coverage offered under the Turkish Public Health System (Nazli et al., 2022). A recent study of Turkish infectious disease physicians described their knowledge of PrEP as “low” (Cimen et al., 2020); while, a recent large-scale survey of Turkish MSM found that both awareness and willingness to use PrEP is quite high (Nazli et al., 2022). The proportion of the population at risk for HIV that would be willing to use PrEP, if made available, is sometimes referred to as “the PrEP gap” (Hayes et al., 2019). Among MSM in Russia and Türkiye, the PrEP gap remains substantial. According to Hayes et al. (2019), more than 45% of MSM at risk for HIV in Russia and Türkiye would be willing to use PrEP if they had access to it. Despite the significant PrEP gap among Russian MSM (Cohen, 2018), PrEP is not readily available in Russia. While there are reports of several PrEP access centers within Russian borders and generic PrEP medications are commercially available at Russian pharmacies, the cost of the medication—in addition to limited PrEP prescribers—contributes to the lack of PrEP availability in Russia (Balayan et al., 2020).

### Research Questions

RQ #1: Did self-reported access to non-pharmacologic HIV prevention methods such as condoms and lubricants differ between sex workers and non-sex workers in France, Russia, or Türkiye?

RQ #2: Did self-reported access to pharmacologic HIV prevention methods (e.g., PrEP) differ between sex workers and non-sex workers in France, Russia, or Türkiye?

## Method

### Participants

We conducted a secondary analysis of the COVID-19 Disparities Study originally administered by Santos et al. (2022). This Internet survey was administered to members of the MSM community via Hornet, a geosocial networking application used by more than 35 million MSM globally. Data were collected between October 25, 2020, and November 19, 2020. Hornet users were invited to participate in a 120-item survey intended to measure the impact of COVID-19 on members of the global MSM community in terms of: economic vulnerability, mental health status, as well as perceived access to HIV prevention, testing, and treatment. At the conclusion of data collection, a total of 21,928 MSM respondents had completed the survey.

Of the 17,520 male-identifying individuals assigned male sex at birth in this sample, the three largest groups of respondents were drawn from France (*n* = 1269), Russia (*n* = 3882), and Türkiye (*n* = 3141). In total, MSM respondents from these three countries comprised 37.8% of the remaining sample (*N* = 8292) and were therefore selected as the focus of this secondary analysis.

### Measures

For each of the study variables below, we describe how investigators operationalized each of the study variables in the COVID-19 disparities survey. Where applicable, we describe our process(es) for re-coding variables prior to performing our analyses.

- **Age–**Because we expect that the relationship between age and PrEP use is nonlinear and likely varies between the sub-populations represented in this data set, we binned this continuous variable into four categories: 18–25 years old, 26–35 years old, 36–45 years-old, and 46 + years old.
- **Sex Work–**Sex worker status was represented as a binary predictor based on whether or not the survey respondent self-identified as a sex worker at the time of survey completion.
- **PrEP Status–**The original survey corresponding to this variable contained seven response options, including: no PrEP use, current PrEP use, PrEP discontinued, PrEP discontinued due to COVID-19, PrEP discontinued as a result of HIV acquisition, “I don’t know,” and “I cannot or do not wish to answer this question.” For the purposes of this secondary analysis, we re-coded this variable into a categorical predictor with three possible outcomes: never used PrEP, discontinued PrEP, and current PrEP use.
- **Education**–The original survey corresponding to this variable contained seven response options, including: no formal education, elementary/primary school, high school/secondary school, trade school/vocational training/apprenticeship, postsecondary education or some college, university—first degree, university—masters/doctorate/post-doctorate. We re-coded this variable into a categorical predictor with four possible outcomes: less than high school, high school, some college/trade school, and college graduate.
- **Employment–**The original survey item corresponding to this variable contained nine response options: employed—full time, employed—part-time, on paid leave, unemployed—lost job due to COVID-19, unemployed prior to the COVID-19 pandemic, student, retired, disabled, and “other.” We re-coded this variable into a categorical predictor with four possible outcomes: employed/on paid leave, unemployed, student, and “other.”
- **Place of Residence–**The original survey item corresponding to this variable contained six response options: a capital city, a large city, a suburb near a large city, a small city/town, a rural area/village, and a farm/isolated house. We re-coded this variable into a categorical predictor with three possible outcomes: large city (e.g., capital city/large city), suburb/small city/town, and rural village/farm.
- **Sexual Orientation—**The original survey item corresponding to this variable contained ten response options: gay, lesbian, bisexual, pansexual, queer, heterosexual, asexual, questioning, “I don’t know,” and “I cannot or do not wish to answer this question.” These responses were re-coded (unless otherwise noted) into a variable with four response options: gay, bisexual, don’t know/questioning, and “other.”
- **HIV Prevention Access–**Participants were asked if they felt they had access to condoms and/or lubricants during the COVID-19 pandemic. Response options included: no, yes—but less access, yes—the same access, yes—more access, “I don’t know,” and “I cannot or do not wish to answer this question. We re-coded this variable into a binary predictor indicating: no access/reduced access to condoms and/or lubricant versus same/more access to condoms and/or lubricant.
- **Ethnic Minority Status**–For respondents outside of the United States, the original survey asked respondents if they self-identified as a racial or ethnic minority. No further distinction was made about racial or ethnic identity. We made no changes to this binary predictor variable in the forthcoming models.

### Statistical Analyses

We first used chi-square tests to determine if there were differences in the proportion of respondents who reported access to condoms and/or lubricant during the COVID-19 pandemic based on self-reported sex worker status. We repeated this process by comparing the proportions of the population respondents who reported alterations in PrEP access during the COVID-19 pandemic based on self-reported sex worker status.

To further elucidate the relationship between the factors associated with self-reported reduced access to condoms and lubricants based on the outcome of our bivariate tests, we conducted a multivariate logistic regression model to further characterize the relationship between access to condoms and lubricants and a series of predictors including sex worker status, age, racial/ethnic minority status, employment status, educational attainment, and place of residence.

To further elucidate the relationship between the factors associated with self-reported PrEP access based on the outcome of our bivariate tests, we used a multinomial logistic regression model to examine the association between self-reported sex worker status, age, racial/ethnic minority status, and place of residence with PrEP use. Multinomial logistic regression is an appropriate statistical method because our primary outcome of interest is a categorical variable represented by one of three levels (never used PrEP, current PrEP use, PrEP discontinued).

## Results

### Sample Description

A total of 8,292 respondents were included in the study from the three countries selected for inclusion in this analysis: France (*n* = 1269), Türkiye (*n* = 3141) and the Russian Federation (*n* = 3882). An overview of the sample characteristics is shown in Table 4. The mean age of respondents from all three countries was 34.39 years (*SD* = 10.44). Respondents were, on average, older in France (*M* = 41.62 years, *SD* = 11.67) than in Türkiye (*M* = 32.85 years; *SD* = 10.18) or Russia (*M* = 33.28 years, *SD* = 9.21). Survey respondents were predominantly located in capital cities or large cities in all three included countries. Of the 4804 (57.9%) of all participants who responded to the survey, 555 (11.6%) identified as a sex worker: 10.1% of the sample in France, 10.8% of the sample in Türkiye and 12.7% of the sample in Russia. The majority of respondents (71.4%) identified as gay and 18.5% identified as bisexual. Approximately 44% of (*n* = 3662) of respondents were HIV negative, 544 (6.6%) self-reported living with HIV. Notably, about half of the survey respondents did not disclose their HIV serostatus (*n* = 4206).

**Table 4:** Sample sociodemographic characteristics (*N* = 8292)

	France (*n* = 1269)				Russia (*n* = 3882)				Türkiye (*n* = 3141)			
	*n*		%		*n*		%		*n*		%	
*Sex Worker Status*												
Identified as sex worker	83		6.5		287		7.4		185		5.9	
Did not identify as sex worker	739		58.2		1978		51.0		1532		48.8	
Not indicated	822		35.2		1617		41.7		1424		45.3	

	Sex worker		Non-sex worker		Sex worker		Non-sex worker		Sex worker		Non-sex worker	
	*n*	*%*	*n*	*%*	*n*	*%*	*n*	*%*	*n*	*%*	*n*	*%*
*Age*												
18–25	9	10.8	56	7.6	81	28.2	373	18.9	92	49.7	360	23.5
26–35	23	27.7	158	21.4	116	40.4	718	36.3	58	31.4	579	37.8
36–45	20	24.1	205	27.7	68	23.7	638	32.3	25	13.5	389	25.4
46 +	31	37.3	320	43.3	22	7.7	249	12.6	10	5.4	204	13.3
*Racial/Ethnic Minority*												
Yes	9	10.8	73	9.9	33	11.5	178	9.0	45	24.3	264	17.2
No	67	80.7	634	85.8	231	80.55	1665	84.2	116	62.7	1077	70.3
Missing	7	8.4	32	4.3	23	8.0	135	6.8	24	13.0	191	12.5
*Sexual Orientation*												
Gay	73	88.0	637	86.2	208	72.5	1494	75.5	125	67.6	1016	66.30
Bisexual	6	7.2	73	9.9	52	18.1	338	17.1	31	16.8	326	21.3
Straight	1	1.2	0	0	1	0.3	16	0.8	2	1.1	38	2.5
Don’t know/Questioning	1	1.2	13	1.8	7	2.4	38	1.9	14	7.6	63	4.1
Other	2	2.4	12	1.6	17	5.9	67	3.4	7	3.8	51	3.2
Missing	0	0	4	0.5	2	0.7	25	1.3	6	3.2	38	2.5
*Place of Residence*												
Large/capital city	39	47.0	321	43.4	218	76.0	1512	76.4	145	78.4	1224	79.9
Town/suburb	32	38.6	280	37.9	59	20.6	425	21.5	31	16.8	257	16.8
Rural/farm	12	14.5	138	18.7	10	3.5	39	2	8	4.3	49	3.2
Missing	0	0	0	0	0	0	2	0.1	1	0.5	2	0.1
*Education*												
Less than secondary	1	1.2	6	0.8	7	2.4	40	2.0	11	5.9	51	3.3
Secondary	17	20.5	89	12.0	47	16.4	222	11.2	66	35.7	375	24.5
Some third level	25	30.1	203	27.5	65	22.6	349	17.6	24	13.0	160	10.4
Graduate	40	48.2	441	59.7	167	58.2	1362	68.9	83	44.9	945	61.7
Missing	0	0	0	0	1	0.3	5	0.3	1	0.5	1	0.1
*Employment Status*												
Employed/Paid Leave	49	59.0	557	75.4	213	74.2	1566	79.2	101	54.6	1013	66.1
Unemployed	20	24.1	72	9.7	30	10.5	176	8.9	34	18.4	172	11.2
Student	3	3.6	21	2.8	25	8.7	119	6.0	36	19.5	177	11.6
Other	11	13.3	89	12	18	6.3	111	5.6	14	7.6	164	10.7
Missing	0	0	0	0	1	0.3	6	0.3	0	0	6	0.4
*PrEP*												
Never used PrEP	53	63.9	596	80.6	252	87.8	1793	90.6	156	84.3	1354	88.4
Current PrEP use	20	24.1	105	14.2	11	3.8	54	2.7	8	4.3	58	3.8
Prior PrEP use	7	8.4	32	4.3	10	3.5	54	2.7	8	4.3	22	1.4
Don’t know/Decline	2	2.4	6	0.8	14	4.9	76	3.8	13	7.0	96	6.3
Missing	1	1.2	0	0	0	0	1	0.1	0	0	2	0.1
*HIV Status*												
HIV negative	58	69.9	592	80.1	187	65.2	1594	76.0	105	56.8	991	64.7
HIV positive	17	20.5	57	7.7	55	19.2	219	11.1	25	13.5	106	6.9
Missing	8	9.6	90	12.2	45	15.7	255	12.9	55	29.7	435	28.4

Among the 5175 (62.4%) participants who elected to disclose their PrEP status, nearly 87% (n = 4498) of survey respondents had never taken PrEP, whereas just over 5% of survey respondents (n = 277) reported current PrEP use. Rates of current PrEP use varied considerably between the three countries, ranging from 1.9% of respondents from the Russian Federation, 2.3% of respondents in Türkiye, and more than 10% of respondents from France.

Findings from Independent samples t tests comparing the mean reported ages of self-identified sex workers versus non-sex workers suggest there was no statistically significant difference in the average age of sex workers (*M* = 40.6, *SD* = 12.13) versus non-sex workers (*M* = 42.6, *SD* = 11.38) in France. However, in Türkiye, respondents who self-identified as sex workers (*M* = 28.6, *SD* = 8.77) were, on average, about two years older than their non-sex worker peers (*M* = 33.8, *SD* = 10.06, *p* < 0.001). Similarly, among respondents in Russia, self-identified sex workers (*M* = 31.7, *SD* = 8.8) were approximately three years younger than their non-sex worker peers (*M* = 34.7, *SD* = 9.3, *p* < 0.001).

### Perceived Access to Non-Pharmacologic HIV Prevention

The proportion of respondents who reported access to condoms and/or lubricants amidst the COVID-19 pandemic appeared to differ across our two access categories (e.g., no/reduced access to condoms and lubricants vs. same/increased access to condoms and lubricants) based upon sex worker status among respondents in Russia (*χ*^2^(3, *N* = 2158) = 10.71, *p* = 0.001) and France (*χ*^2^(3, *N* = 785) = 15.88, *p* < 0.001), but not Türkiye (*χ*^2^(3, *N* = 1602) = 0.48, *p* = 0.49).

Among respondents in France, as shown in Table 5 below, self-identification as a sex worker was associated with reduced odds of access to condoms and lubricants during the COVID-19 pandemic (OR = 0.39 *p* = 0.003, 95% CI = 0.21–0.73). Other model variables associated with interrupted access to condoms and lubricants among respondents in France were identifying as a member of a racial/ethnic minority group (OR = 0.45, *p* = 0.015, 95% CI = 0.24–0.86) and being a student (OR = 0.21, *p* = 0.022, 95% CI = 0.05–0.8).

**Table 5:** Logistic regression model: Survey respondent characteristics and association with reduced access to condoms and lubricant amidst the COVID-19 pandemic in France (*N* = 748)

	OR	SE	*p value*	*95% CI*
*Sex Work*	0.39	0.12	0.003*	0.21–0.73
*(Reference* = *Non-sex worker)*				
*Age*				
*(Reference* = *18*–*25 Years)*				
26–35 years-old	0.98	0.56	0.972	0.32––3.03
36–45 years-old	0.69	0.39	0.517	0.23–2.12
46 + years old	0.54	0.3	0.268	0.18–1.60
*Racial/Ethnic Minority*	0.45	0.15	0.015*	0.24–0.86
*(Reference* = *Non-minority)*				
*Employment Status*				
*(Reference* = *Employed)*				
Unemployed	0.53	0.18	0.057	0.27–1.02
Student	0.21	0.14	0.022*	0.05–0.80
Other	0.58	0.19	0.105	0.30–1.11
*Educational Attainment*				
*(Reference* = *Less than high school)*				
High School	2.3	2.19	0.38	0.36–14.87
College/Trade School	2.42	2.25	0.339	0.39–14.90
College Graduate	3.64	3.34	0.16	0.60–21.97
*Place of Residence*				
*(Reference* = *Large/Capital City)*				
Suburb/Small Town	0.75	0.2	0.286	0.45–1.27
Rural Village/Farm	0.72	0.23	0.311	0.38–1.36
*χ*^2^(13, *N* = 748) = 33.51, *p* = 0.001)				

^*^Denotes significance at the conventional *p* < .05 level

In Russia, respondents who self-identified as sex workers reported reduced access to condoms and lubricants (OR = 0.54, *p* = 0.07, 95% CI = 0.35–0.85) as compared to their non-sex worker peers, as did those who self-identified as a member of a racial/ethnic minority group (OR = 0.55, *p* = 0.016, 95% CI = 0.34–0.89). As shown in Table 6, additional factors associated with reduced access to condoms and lubricants in Russia were being unemployed (OR = 0.31, *p* < 0.01, 95% CI = 0.20–0.49) and residing in a suburb/small town (OR = 0.63, *p* = 0.027, 95% CI 0.42–0.95).

**Table 6:** Logistic regression model: Survey respondent characteristics and association with reduced access to condoms and lubricant amidst the COVID-19 pandemic in Russia (*N* = 2003)

	OR	SE	*p*-value	95% CI
Sex Work	0.54	0.12	0.007*	0.35–0.85
*(Reference* = *Non-sex worker)*				
Age				
*(Reference* = *18–25 Years)*				
26*–*35 years-old	0.88	0.24	0.638	0.52–1.50
36*–*45 years-old	1.37	0.41	0.297	0.76–2.46
46 + years old	1.41	0.54	0.366	0.67–2.97
Racial/Ethnic Minority	0.55	0.14	0.016*	0.34–0.89
*(Reference* = *Non-minority)*				
Employment Status				
*(Reference* = *Employed)*				
Unemployed	0.31	0.07	0.000*	0.20–0.49
Student	0.64	0.24	0.239	0.30–1.35
Other	2.19	1.33	0.193	0.67–7.19
Educational Attainment				
*(Reference* = *Less than high school)*				
High School	2.10	1.20	0.199	0.68–6.48
College/Trade School	1.54	0.83	0.422	0.54–4.45
College Graduate	1.74	0.90	0.280	0.64–4.78
Place of Residence				
*(Reference* = *Large/Capital City)*				
Suburb/Small Town	0.63	0.13	0.027*	0.42–0.95
Rural Village/Farm	0.58	0.33	0.333	0.20–1.74
*χ*^2^(13, *N* = 2003) = 56.19, *p* < 0.001)				

^*^Denotes significance at the conventional *p* < .05 level

### Perceived Access to Pharmacologic HIV Prevention

The proportion of respondents who reported access to PrEP services during the COVID-19 pandemic appeared to differ across our four PrEP access categories (e.g., never used PrEP, currently using PrEP, stopped PrEP, and “don’t know/decline to respond”) by sex worker status among respondents in France (*χ*^2^(3, *N* = 821) = 12.18, *p* = 0.007) and Türkiye (*χ*^2^(8.46, *N* = 1530) = 0.04, *p* = . 02), but not Russia (*χ*^2^(3, *N* = 1977) = 2.47, *p* = . 48). Given the differences in PrEP access observed between sex workers and non-sex worker respondents in France and Türkiye, we performed two multinomial regression models, one each for France and Türkiye. We excluded respondents who indicated they were living with HIV because these individuals were not PrEP eligible. We also excluded respondents who indicated they were unfamiliar with PrEP and those who indicated that they did not know their PrEP status. Given the small counts in some of the response categories for the sexual orientation, education, and employment status variables presented previously, these predictors were not included in the two forthcoming models.

The relative risk of never using PrEP among survey respondents in France appeared to decrease with increasing age, as evidenced by the lower relative risk ratio in the 36–45 years-old and 46 + years-old age groups (see Table 7). Sex workers were less likely to report never using PrEP, as evidenced by the relative risk ratio of 0.40 (*p* = 0.005, 95% CI = 0.21–0.76). In other words, for sex workers relative to non-sex workers, the relative risk of never using PrEP would be expected to decrease by a factor of 0.40, given all other variables in the model are held constant. Conversely, however, respondents located in suburbs/small towns in France were *more* likely to report never using PrEP as opposed to their urban peers. There were no significant predictors differentiating between the respondents who reported discontinuation of PrEP and active PrEP use in this model.

**Table 7:** Multinomial logistic regression model: Association between sociodemographic characteristics and PrEP use among survey respondents in France (*N* = 705)

Active PrEP Use	RRR	SE	*p*	95% CI
	(Base Outcome)			
*Never Used PrEP*				
Age				
*(Reference* = *18*–*25 Years)*				
26–35 years-old	0.33	0.22	0.091	0.10–1.19
36–45 years-old	0.20	0.12	0.009*	1.49–17.22
46 + years old	0.26	0.16	0.028*	1.16–13.08
Racial/Ethnic Minority	1.20	0.42	0.607	0.60–2.40
*(Reference* = *Non-minority)*				
Sex Work	0.40	0.13	0.005*	0.21–0.76
*(Reference* = *Non-sex worker)*				
Place of Residence				
*(Reference* = *Large/Capital City)*				
Suburb/Small Town	1.86	0.44	0.010*	1.16–2.97
Rural Village/Farm	1.47	0.43	0.188	0.83–2.61
*PrEP Discontinuation*				
Age				
*(Reference* = *18*–*25 Years)*				
26–35 years-old	0.62	0.62	0.635	0.09–4.40
36–45 years-old	0.14	0.15	0.066	0.79–21.74
46 + years old	0.55	0.53	0.535	0.10–12.74
Racial/Ethnic Minority	0.95	0.66	0.938	0.24–3.68
*(Reference* = *Non-minority)*				
Sex Work	0.99	0.56	0.979	0.33–2.98
*(Reference* = *Non-sex worker)*				
Place of Residence				
*(Reference* = *Large/Capital City)*				
Suburb/Small Town	0.90	0.40	0.807	0.37–2.16
Rural Village/Farm	0.48	0.33	0.283	0.13–1.82

^*^Denotes significance at the conventional *p* < .05 level

As shown in Table 8, the relative risk of never having used PrEP among survey respondents in Türkiye also appeared to decrease with increasing age, as evidenced by the decrease in the relative risk ratio in both the 26–35 and 36–45 age group categories. Sex worker status did not appear significant in terms of differentiating between individuals who use/have used PrEP, discontinued PrEP, and/or never heard of PrEP. For individuals in suburbs/small towns relative to urban settings, the relative risk of discontinuing PrEP versus continuing PrEP use would be expected to increase by a factor of 7.82 (*p* = 0.017, 95% CI 1.44–42.57) given that all other variables in the model are held constant. This finding suggests that individuals in suburbs/small towns in Türkiye may be more likely than urban survey respondents to report discontinuation of PrEP versus active PrEP use during the COVID-19 pandemic.

**Table 8:** Multinomial logistic regression model: Association between sociodemographic characteristics and PrEP use among survey respondents in Türkiye (*N* = 1309)

Active PrEP use	RRR	SE	*p*	95% CI
	(Base Outcome)			
*Never Used PrEP*				
Age				
*(Reference* = *18–25 Years)*				
26*–*35 years-old	0.17	0.13	0.021*	0.38*–*0.76
36*–*45 years-old	0.21	0.17	0.048*	0.43*–*0.99
46 + years old	0.34	0.32	0.251	0.06*–*2.13
Racial/Ethnic Minority	3.35	2.47	0.101	0.79*–*14.23
*(Reference* = *Non-minority)*				
Sex Work	0.96	0.72	0.956	0.22*–*4.20
*(Reference* = *Non-sex worker)*				
Place of Residence				
*(Reference* = *Large/Capital City)*				
Suburb/Small Town	2.60	1.93	0.197	0.61*–*11.12
Rural Village/Farm	0.41	0.31	0.242	0.09*–*1.83
*PrEP Discontinuation*				
Age				
*(Reference* = *18–25 Years)*				
26*–*35 years-old	1.07	1.48	0.447	0.38*–*8.91
36*–*45 years-old	0.86	1.00	0.898	0.09*–*8.30
46 + years old	0.38	0.59	0.534	0.02*–*7.94
Racial/Ethnic Minority	3.79	3.40	0.138	0.65*–*22.01
*(Reference* = *Non-minority)*				
Sex Work	2.63	2.48	0.302	0.42*–*16.69
*(Reference* = *Non-sex worker)*				
Place of Residence				
*(Reference* = *Large/Capital City)*				
Suburb/Small Town	7.82	6.76	0.017*	1.44*–*42.57
Rural Village/Farm	1.00	1.28	0.997	0.08*–*12.31

^*^Denotes significance at the conventional *p* < .05 level

## Discussion

Findings from this secondary analysis highlight important differences between men who have sex with men sex workers (MSMSW) and MSM not engaged in SW in access to both non-pharmacologic and pharmacologic HIV prevention methods during the COVID-19 pandemic in France, Russia, and Türkiye. It is concerning that marginalized groups—such as sex workers—reported a lack of access to condoms and lubricant during the COVID-19 pandemic in France and Russia. However, this observation is consistent with other studies noting that MSM who engage in sex work in other jurisdictions reported interrupted access to sexual health resources as a result of the COVID-19 pandemic (Callander et al., 2022; McBride et al., 2023). MSM who engage in sex work may have been more likely to engage in sexual activity during the pandemic as they were economically dependent on such activity. The absence of sexual health services and/or the unavailability of non-pharmacologic HIV/STI prevention methods like condoms and lubricants may have been more noticeable among sex workers. Given the social climate in France, MSM sex workers in France likely had greater access to condoms and lubricants prior to the pandemic and were more disproportionately affected by lockdowns and shelter-in-place orders. Despite comparatively fewer lockdowns, MSM sex workers in Russia and/or Türkiye likely experienced reduced access to HIV prevention practices prior to the pandemic because of the social climate and attitudes toward sex work and same-sex behaviors in these countries. Furthermore, the reductions in access to condoms and lubricant reported among MSM sex workers in Russia could reflect reduced humanitarian aid and non-profit contributions to sexual health efforts in Russia during the height of the COVID-19 pandemic.

We also found that a larger proportion of MSM participants in France who identified as sex workers endorsed PrEP use as compared to their non-sex worker peers. Additionally, among the respondents in France who self-identified as sex workers, there was a significantly lower likelihood of PrEP discontinuation as compared to their non-sex worker peers. Taken together, these findings suggest that the MSM sex workers from France in this sample were more likely to continue using and/or seeking out PrEP during the COVID-19 pandemic. This finding is reassuring because it suggests that MSMSW who are at heightened risk for acquiring HIV in France continued to access effective HIV prevention tools during the COVID-19 pandemic. As described in a study of U.S. MSM who engage in sex work conducted by Sundelson et al. (2022), it is possible that this finding among MSMSW in France reflects greater PrEP awareness among sex workers. As a result of this increased awareness of PrEP, sex workers may perceive easier access to PrEP. Furthermore, this finding may reflect a heightened awareness of HIV as a potential occupational hazard among members of the MSM sex work community.

Our analyses also found several additional sociodemographic factors were associated with reduced access to condoms and lubricants in both France and Russia. For example, self-identification as a member of a racial or ethnic minority group was associated with reduced access to condoms and lubricants in both countries. In Russia, self-identification as a student was associated with reduced access to these HIV prevention strategies. In France, individuals who endorsed being unemployed and residing in a suburb were associated with reduced access to condoms and lubricants. These findings may help in targeting specific groups for HIV prevention efforts, particularly where uptake of HIV PrEP remains low.

In jurisdictions where access to HIV PrEP is established (France) or evolving (Türkiye), self-reported PrEP access appeared more common among slightly older MSM respondents (e.g., older than the reference 18–21 year-old age group) in our logistic regression models. These findings are consistent with other communities with increased/increasing PrEP uptake, suggesting that the youngest MSM community members, who are perhaps at highest risk of HIV acquisition, remain less likely to use PrEP (Sullivan et al., 2020).

The absence of a statistically significant difference in PrEP access between sex workers and non-sex workers in Russia could be explained by multiple factors. This lack of difference is likely attributable to a widespread absence of PrEP availability for HIV prevention, which could be attributed to Russian attitudes toward MSM behavior and homosexuality. Also troubling, the lack of PrEP access in more rural/less urban settings observed in the models in this analysis is consistent with findings from other global studies of PrEP use (ECDC, 2021; Siegler et al., 2019). Over the past ten years, the implementation of PrEP has been more prominent in urban areas, possibly due to a higher concentration of MSM community members. The greater likelihood of suburban MSM in Türkiye discontinuing PrEP might be attributed to reduced accessibility during the pandemic. The absence of a similar trend among participants in France might suggest differences in PrEP delivery between the two countries. Further, these differences could involve the availability of PrEP prescribers and/or lower levels of PrEP-related stigma in France compared to Türkiye.

This study has several limitations. The findings of this study are based on a sample of survey respondents recruited from a commonly used MSM geosocial networking application. While the use of such applications is common among MSM, the findings may be less generalizable to individuals who do not use such applications. It is also possible that the implementation of COVID-19 polices varied within different regions of the same country, and this variation cannot be accounted for in our models based on the data available. While this survey was anonymous, social desirability bias and/or societal stigma could have influenced participants’ self-reported survey responses and potentially translate to an underestimation of the associations presented.

### Conclusion

This secondary analysis examined access to HIV prevention and treatment in France, Türkiye, and the Russian Federation in the context of the COVID-19 pandemic, with a particular focus on MSM sex workers—a group historically underrepresented in occupational and public health research. Taken together, the observations from this study provide valuable insights that could inform HIV prevention and outreach efforts during a future public health emergency. Perhaps most importantly, the findings from this study highlight the need for improved access to HIV prevention measures among both MSM sex workers and the MSM community both during and in the wake of the global COVID-19 pandemic. Further research is necessary to examine PrEP uptake in the post-COVID era now that most global healthcare systems have largely recovered from the acute strain of the COVID-19 pandemic and global PrEP awareness continues to increase. Emerging HIV prevention strategies—including long-acting injectable PrEP (LAI PrEP)—could positively affect PrEP uptake among MSM sex workers and the MSM population at large. Additional research is necessary to determine how the global MSM community will respond to LAI PrEP.

In this study, we identified multiple significant disparities in access to HIV prevention services for MSM living in the three surveyed nations. This study highlights the HIV prevention challenges affecting MSM sex workers during the COVID-19 pandemic. Based on our findings, ensuring uninterrupted access to condoms and lubricants is likely an important and potentially high-yield intervention in jurisdictions where PrEP is not yet widely available. The reduced access to condoms and lubricants seen among respondents who self-identified as racial or ethnic minorities should also inform non-PrEP HIV prevention efforts. Providers and public health officials should work with local community groups to ensure unfettered access to condoms and lubricant and that these products are being offered to community members in a culturally appropriate manner.

There are reports of all three study countries adopting telehealth practices to provide healthcare services (Agaoglu & Karagoz, 2024; Lagutin et al., 2023; Yaghobian et al., 2020). Despite this, there are no recent reports of telehealth services being leveraged to provide sexual health services in any of the study countries. Mail-order and/or telehealth interventions may interest providers and public health officials as part of an expanded outreach effort in jurisdictions where the social climate might allow for such interventions.

Our findings cast a wider light on access to HIV prevention during the COVID-19 pandemic. For younger MSM and MSM living outside of major urban centers, there are MSM who would benefit from increased attention under existing PrEP initiatives. While PrEP access is likely stymied in Russia, given the nation’s anti-LGBT policy stance, PrEP appears to be gaining traction among MSM and healthcare providers in Türkiye. In both Türkiye (where PrEP programs are evolving) and in France (where PrEP uptake is more commonplace), providers and public health officials should consider how to ensure that young MSM and MSM outside of major urban hubs are included in outreach and education efforts. Moreover, providers and public health officials should develop contingency plans to successfully navigate shifting public health priorities during future public health emergencies to ensure continuity of access to valuable HIV prevention tools for those who need them most.
